# Deep Learning and Multiplex Networks for Accurate Modeling of Brain Age

**DOI:** 10.3389/fnagi.2019.00115

**Published:** 2019-05-22

**Authors:** Nicola Amoroso, Marianna La Rocca, Loredana Bellantuono, Domenico Diacono, Annarita Fanizzi, Eufemia Lella, Angela Lombardi, Tommaso Maggipinto, Alfonso Monaco, Sabina Tangaro, Roberto Bellotti

**Affiliations:** ^1^Dipartimento Interateneo di Fisica “M. Merlin”, Università degli studi di Bari “A. Moro”, Bari, Italy; ^2^Istituto Nazionale di Fisica Nucleare, Bari, Italy; ^3^Laboratory of Neuro Imaging, USC Stevens Neuroimaging and Informatics Institute, Keck School of Medicine of USC, University of Southern California, Los Angeles, CA, United States; ^4^Istituto Tumori “Giovanni Paolo II” - I.R.C.C.S., Bari, Italy

**Keywords:** age prediction, brain, deep learning, lifespan, aging, structural MRI, machine learning, multiplex networks

## Abstract

Recent works have extensively investigated the possibility to predict brain aging from T1-weighted MRI brain scans. The main purposes of these studies are the investigation of subject-specific aging mechanisms and the development of accurate models for age prediction. Deviations between predicted and chronological age are known to occur in several neurodegenerative diseases; as a consequence, reaching higher levels of age prediction accuracy is of paramount importance to develop diagnostic tools. In this work, we propose a novel complex network model for brain based on segmenting T1-weighted MRI scans in rectangular boxes, called patches, and measuring pairwise similarities using Pearson's correlation to define a subject-specific network. We fed a deep neural network with nodal metrics, evaluating both the intensity and the uniformity of connections, to predict subjects' ages. Our model reaches high accuracies which compare favorably with state-of-the-art approaches. We observe that the complex relationships involved in this brain description cannot be accurately modeled with standard machine learning approaches, such as Ridge and Lasso regression, Random Forest, and Support Vector Machines, instead a deep neural network has to be used.

## Introduction

Recently, neuroimaging approaches predicting brain aging have received an increasing attention, especially thanks to the design and development of extremely accurate strategies (Franke et al., 2010; Cole et al., 2017a,b). In fact, the possibility of relying on accurate age predictions allows, as a consequence, the definition of age-related biomarkers for the early detection of anomalous or pathological conditions (Dosenbach et al., 2010; Franke et al., 2012). In particular, machine learning models have been used to learn the aging trajectories of healthy brains thus yielding two main results (Cole and Franke, 2017): (i) predicted age can differ from the actual one and this difference and its entity can suitable define a marker for anomalous/pathological aging (Dukart et al., 2011; Koutsouleris et al., 2013); (ii) subject-specific aging processes can be learned, thus driving personalized monitoring or treatment (when needed) (Baker and Martin, 1997; Cole et al., 2018).

The effectiveness of machine learning methods has resulted to be almost ubiquitous (Hung et al., 2006; Zacharaki et al., 2009; Abraham et al., 2014; Khedher et al., 2015; Al Zoubi et al., 2018). Computer aided detection systems for accurate detection of brain diseases have been thoroughly investigated, nevertheless there are several studies, for example about Alzheimer's disease, suggesting there is still room for significant improvement (Bron et al., 2015; Amoroso et al., 2018a; Raḿırez et al., 2018). More recently, promising results toward these desirable improvements have been found in two distinct directions. On one hand, brain connectivity: describing the brain as a complex network and investigating its properties would enhance the possibility of detection for anomalies and pathological conditions affecting the normal functioning of the brain (Dyrba et al., 2015; Amoroso et al., 2018c); on the other hand, deep learning: the adoption of deep learning techniques, prompted by an increment of both computational resources and observations available to run the learning processes, has become a prominent choice for analyzing medical images for disparate uses, such as segmentation, registration, and classification (Ortiz et al., 2016; Litjens et al., 2017; Shen et al., 2017).

In this work, we present an attempt to combine complex network framework and deep learning strategies to provide a novel accurate modeling of brain age. In particular, we use a multiplex network, which is a multi-layer network. A multiplex is a network with many layers, each of one representing a single subject; the nodes are brain anatomical districts and the connections are their pairwise similarities (Kivelä et al., 2014). Recent studies have demonstrated the advantage of considering multiplex networks instead of single networks in terms of intrinsic information: actually, the information content of the multiplex is not just the sum of the information content of its layers (Battiston et al., 2014; Menichetti et al., 2014).

As for standard networks, multiplex networks can be characterized by suitable metrics (Nicosia and Latora, 2015; Estrada, 2018); in particular, we use nodal properties to obtain a feature representation of a brain and then use this framework to feed a deep learning model to predict the brain age. We compare the performance of deep learning with state-of-the-art regression strategies, such as Lasso regression, Ridge regression, Support Vector Machine, and Random Forest regressions. Besides, we identify the brain regions which seem to majorally affect the age prediction.

## Materials and Methods

###  Image Processing

In this work we use data from 5 publicly available sources: ABIDE^1^ (Autism Brain Imaging Data Exchange), ADNI^2^ (Alzheimer's Disease Neuroimaging Initiative), Beijing Normal University^3^, ICBM^4^ (International Consortium for Brain Mapping), and IXI^5^ (Information eXtraction from Images).

We selected a dataset including 484 subjects in order to obtain a roughly uniform distribution in the age range 7−80 years; in particular 133 subjects ranged from 7 to 20 years, 120 from 20 to 40 years, 127 from 40 to 60 years, and 104 above 60 years, see Supplementary Materials for further details. Subjects whitin the 0−7 age range are not included in this study because, as better explained in the Discussion section, they require specific image processing techniques which are not require for the age ranges considered here, instead.

Mean age was 37.3±20.4 years. All neuroimaging data used in this study were T1-weighted MPRAGE brain scans (1.5 *T* or 3.0 *T*); 1.5 *T* and 3.0 *T* scans do not significantly differ in their power to detect gray matter changes (Ho et al., 2010). The participants were healthy controls, thus excluding the presence of neurodegenerative or psychiatric diseases.

Brain scans were normalized in intensity and skull-stripped using the Brain Extraction Tool from the FSL library (Jenkinson et al., 2005); then, non-linear registration was performed using the Advanced Normalization Tools pipeline (Avants et al., 2009) to the MNI152 template; accordingly, all registered scans resulted in 1 × 1 × 1 *mm*^3^ resolution so that, from now onward, voxels and *mm*^3^ will be interchangeably used.

**Figure 1:**
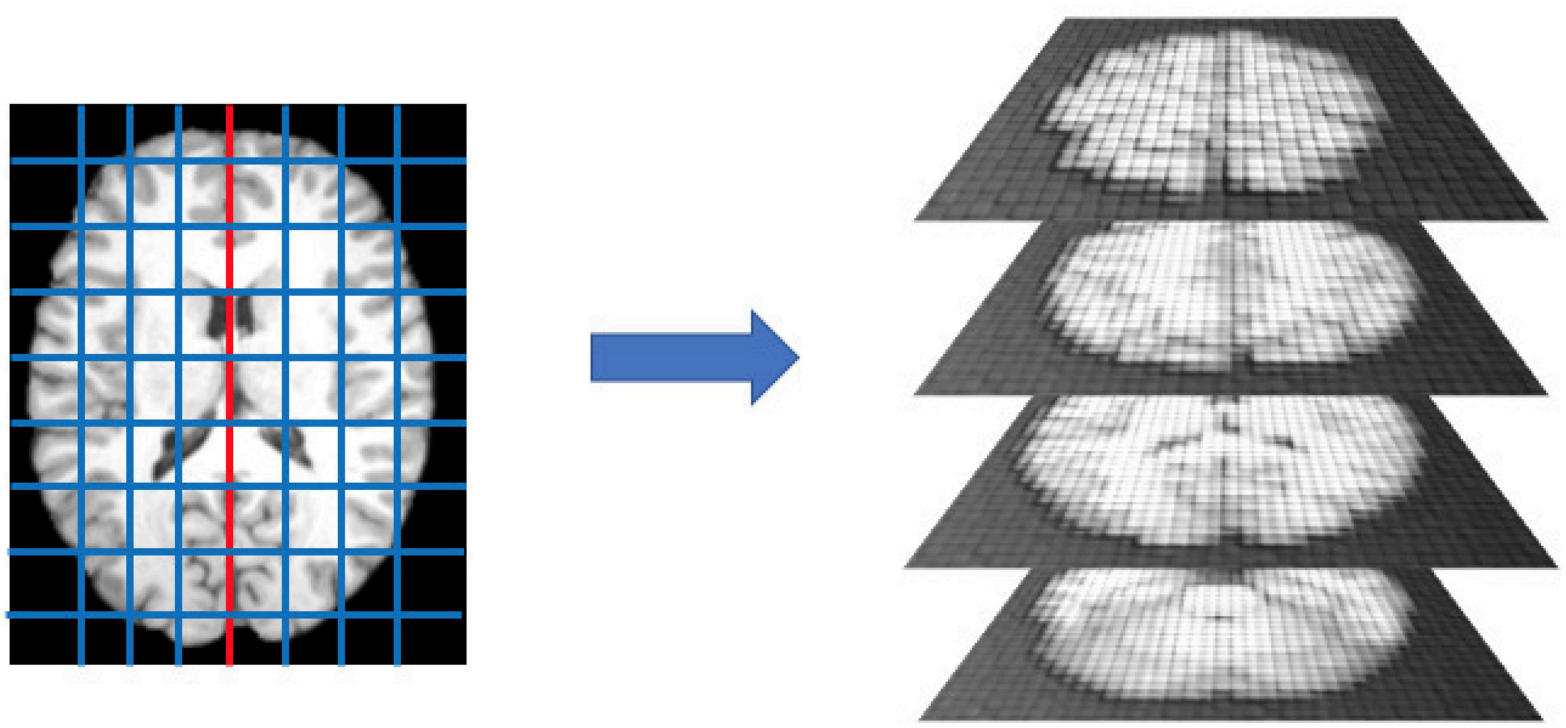
After dividing the brain into left and right hemispheres, each hemisphere is divided in 300 patches. This operation is performed for each subject in the cohort, after registration, thus each patch is expected to roughly contain the same anatomical district and analogous distributions of white matter, gray matter, and cerebrospinal fluid.

After spatial normalization we separated the left and the right brain hemispheres and segmented each part in rectangular boxes, called patches, of *l*_1_×*l*_2_×*l*_3_ dimensions. A schematic representation is provided by Figure 1.

According to a previous study about neurodegenerative processes in Alzheimer's disease (Amoroso et al., 2018b), we used *l*_1_ = 10, *l*_2_ = 15, and *l*_3_ = 20 with *l*_1_, *l*_2_, and *l*_3_ lengths, in voxels, along the coronal, the axial, and the sagittal orientations, respectively. Thus, each subject's brain was represented by a collection of 600 patches.

###  The Network Model

By definition, a complex network *G* = *G*(*N, L*) is a couple of two distinct sets (Boccaletti et al., 2006): *N*, the set of nodes, and *L*, the set of links. The nodes are the elements of the system one wants to model while the links represent the interactions among them. This basic framework does not take into account the entity of the interactions; to consider this aspect weighted networks are introduced (Newman, 2004). Weighted networks are assigned a third set of elements *W* whose elements *w*_*ij*_, called weights, represent the strengths of each interaction between the nodes *i* and *j*; the weights are usually real or integer numbers, so that a weighted network is denoted *G* = *G*(*N, L, W*).

In this work, the brain networks are defined using each patch as a node. Patches consist of 3, 000 voxels whose intensity gray levels ranges from 0 to 1. Accordingly, the whole brain is segmented in 600 patches. We considered each patch as a vector with 3, 000 components and measured the Pearson's correlation between each pair of vectors thus obtaining the pairwise similarities, thus we built a weighted network whose nodes were the patches and whose weights were given by the measured correlations. Pearson's correlations range from −1 to 1, however to take into account the left/right symmetry of the brain we kept the absolute value of correlations. Accordingly, our networks consist of 600 × 600 symmetric adjacency matrices whose rows and columns represent the brain patches and whose elements, ranging from 0 to 1, their absolute Pearson's correlations. It is worth noting that the brain network used in this work is mathematical, in fact nodes have no direct anatomical counterparts and edges are correlations, a mathematical similarity metric.

Once the single network representation was obtained for each subject, we built a multiplex model, i.e., a network composed by several layers, in which the same number of nodes can be connected in different ways (Nicosia et al., 2013). Usually, when building a multiplex model, nodes remain unchanged, what changes is the nature of links: for example, in transport networks, the nodes could be the neighbors of a city and the layers the types of transport considered (routes, trains, …). Age shapes brain networks by modifying the spatial distribution of white matter, gray matter, and cerebrospinal fluid and, therefore, the way brain regions are connected, i.e., their pairwise similarity. Accordingly, it is natural to define a different layer α for each age and, thus, for each subject.

Finally, we measured some specific nodal metrics to characterize the multiplex model. Specifically, we considered the following features:

- Strength *s*. The sum of the weights associated to the connections of a node is a common centrality metrics used to characterize important nodes within a network. The strength of the node *i* in a layer α is:
  $$s_{i}^{\alpha }=\overset{N}{\underset{j=1}{\sum }}w_{ij}$$
- Inverse Participation *Y*. It is also important to characterize how strengths are distributed within a network in order to understand the relative importance of a node. The inverse participation of the node *i* in a layer α is:
  $$Y_{i}^{\alpha }=\overset{N}{\underset{j=1}{\sum }}{\left(\frac{w_{ij}^{\alpha }}{s_{i}^{\alpha }}\right)}^{2}$$
- Multistrength. The analogous of the strength in a multiplex model.
- Multi-Inverse Participation. The Inverse Participation computed with respect of the multiplex.

Further details, especially about multiplex metrics, are provided for example in Amoroso et al. (2018b). Besides, we computed the conditional probabilities of strength and multistrength against the nodes with degree *k*; conditional strength for degree *k* in the layer α is:

$$s{\left(k\right)}^{\alpha }=\frac{1}{N_{k}}\overset{N}{\underset{i=1}{\sum }}s_{i}^{\alpha }\delta \left(k_{i}^{\alpha },k\right)$$

with *N*_*k*_ the number of nodes with degree *k* and δ being the Kronecker function, which is equal to one only when the nodal degree $k_{i}^{\alpha }$ is *k* and zero otherwise.

Analogously, the conditional mean of inverse participation for degree *k* in the layer α is:

$$Y{\left(k\right)}^{\alpha }=\frac{1}{N_{k}}\overset{N}{\underset{i=1}{\sum }}Y_{i}^{\alpha }\delta \left(k_{i}^{\alpha },k\right)$$

**Figure 2:**
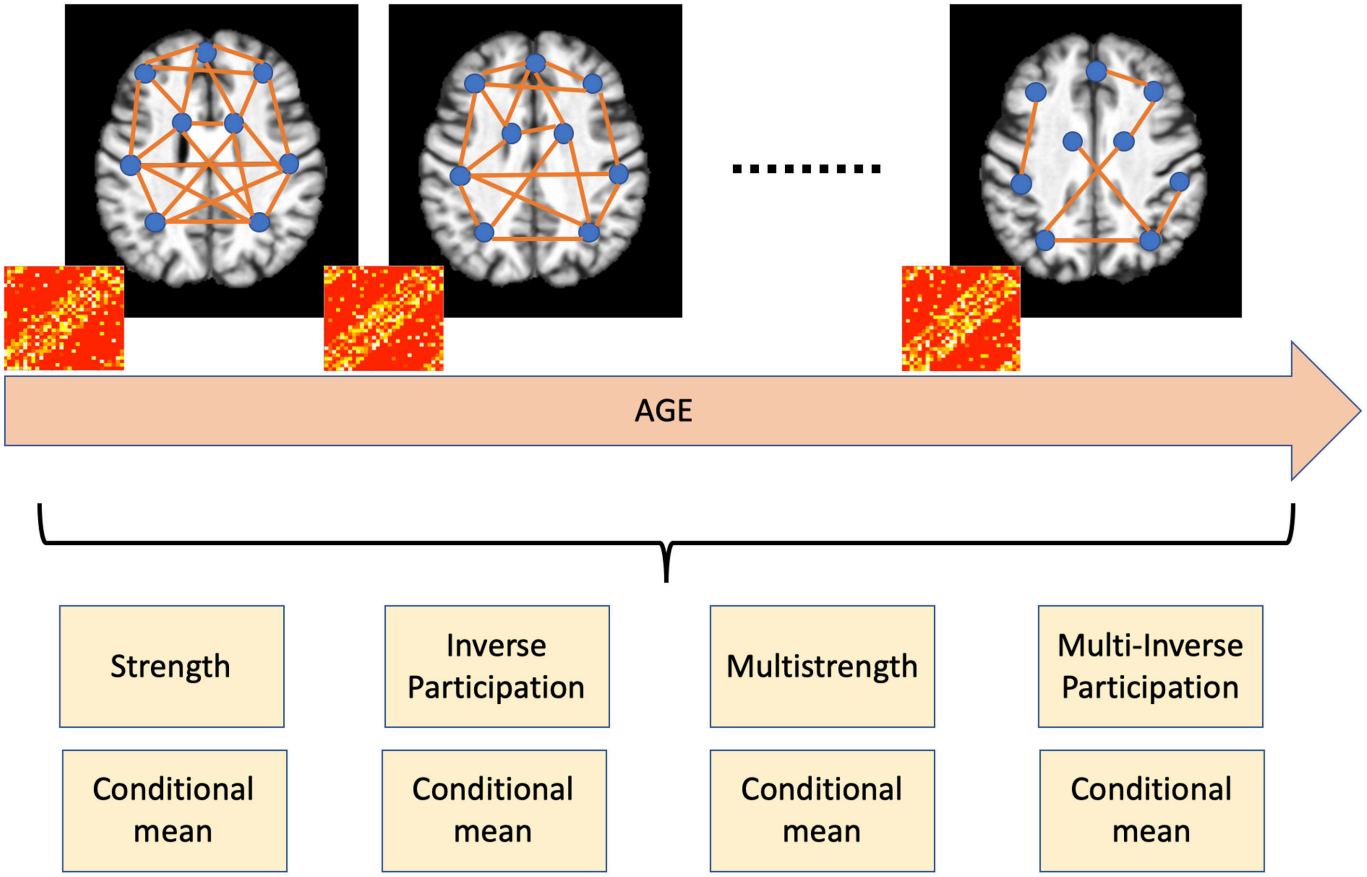
Age shapes brain networks by modifying the spatial distribution of white matter, gray matter, and cerebrospinal fluid. Accordingly, nodal metrics, such as strength and inverse participation, allow the detection and quantification of these age-related changes.

In the end our multiplex representation yielded *M* = 8 × |*N*| features for each subject, with |*N*| being the cardinality of *N*, |*N*| = 600, and, therefore, *M* = 4, 800. The conceptual workflow is presented in Figure 2.

The basic idea behind our approach is that one of the main effects involved by aging is brain atrophy; our framework allows the detection of age related changes in brain using a complex network model and therefore the possibility to yield accurate brain age prediction. Pearson's correlation is a suitable metric to characterize the spatial distribution of white matter, gray matter, and cerebrospinal fluid and the multiplex framework takes into account how this distribution changes over time; besides, the previously mentioned nodal properties measure how these changes affect the networks and the different brain regions, therefore, they allow a direct easy-to-interpret overview of aging effects.

###  Regression

Once we obtained a feature representation for all subjects, we trained our deep learning regression model. To assess the robustness of our brain model and to confirm the effectiveness of deep learning we also evaluated four other different regression models that are widely adopted for their accuracy: Lasso regression, Ridge regression, Support Vector Machine, and Random Forest. The presented results were cross-validated with a 10-fold procedure repeated 100 times. To evaluate the regression performance we adopted three different metrics:

- Mean Absolute Error (MAE).
  $$\text{MAE}=\frac{1}{S}\overset{S}{\underset{i=1}{\sum }}|y_{i}-\hat{y_{i}}|;$$
- Root Mean Squared Error (RMSE).
  $$\text{RMSE}=\sqrt{\frac{1}{S}\overset{S}{\underset{i=1}{\sum }}{\left(y_{i}-\hat{y_{i}}\right)}^{2}};$$
- Pearson's correlation (ρ).
  $$\rho =\frac{\sum_{i=1}^{S}\left(y_{i}-\bar{y}\right)\left(\hat{y_{i}}-\bar{ŷ}\right)}{\sqrt{\sum_{i=1}^{S}{\left(y_{i}-\bar{y}\right)}^{2}}\sqrt{\sum_{i=1}^{S}{\left(\hat{y_{i}}-\bar{ŷ}\right)}^{2}}}.$$

with *S* being the sample size, *y*_*i*_ the chronological age, $\hat{y_{i}}$ the predicted brain age, $\bar{y}$ the sample average age, and $\bar{ŷ}$ the average brain predicted age. All our models were implemented with the open source R language.

#### Deep Learning

A deep neural network is, by definition, a network with more than two hidden layers (Hinton et al., 2006). Deep learning strategies are designed to learn, thanks to the complex interactions instanced between neural networks' hidden layers, accurate representations of the provided observations; in recent years, deep learning has significantly improved the state-of-the-art in several fields, such as speech recognition, object detection, and diagnosis support systems (LeCun et al., 2015).

Artificial neural networks with few learning layers, also called shallow networks, have been known for decades; since the introduction of backpropagation algorithms, their training has shown very promising perspectives but raised several feasibility issues, especially for the exponential growth of computational requirements. Besides, a theorem stating that multilayer feed forward networks with a sufficient number of neurons and as few as one hidden layers are universal approximators, strongly suggested to invest more effort on simpler architectures than deeper ones (Hornik et al., 1989). Finally, there was a common belief that deep neural network learning algorithms (especially the gradient descent) could be trapped in local minima preventing the possibility to yield stable and accurate results.

Recent results, both theoretical and empirical, showed that these issues can be overcome and deep learning algorithms can achieve unmatched performances in several domains. Moreover, the possibility to easily access huge computational resources has removed the practical limitations preventing the wide-spread adoption of deep learning strategies.

**Figure 3:**
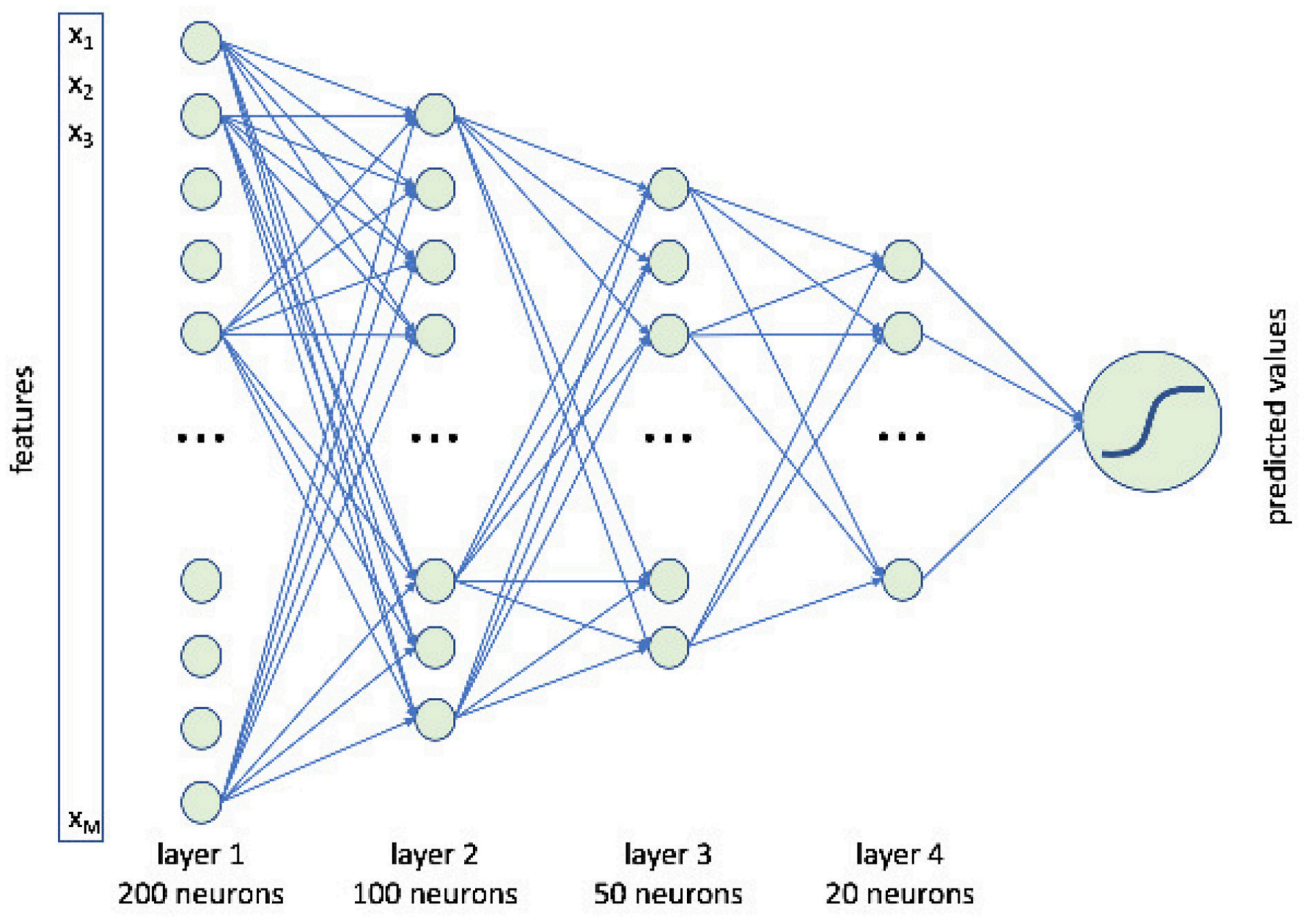
A schematic representation of our deep neural network. It consisted of four hidden layers composed of 200, 100, 50, and 20 neurons.

In this paper, we use a feedforward deep neural networks with four hidden layers respectively including 200, 100, 50, and 20 neurons, see Figure 3.

This architecture was implemented with the “h2o” R package. Among the possible tuning parameters, besides the number of hidden layers with the corresponding neurons, this package offers the possibility to define:

- activation functions including: hyperbolic tangent, linear rectifier, and maxout;
- learning rate;
- training epochs;
- regularization (*L*_1_ or *L*_2_);
- tolerance;
- rate decay.

The flexibility offered by deep learning architectures is also their major drawback, as tuning these models can be challenging. This is why another important option provided by the “h2o” package (and many others) is the so called *grid search*, allowing the systematic exploration of the configurations' space, thus automatically determining the most effective design. We explored different numbers of layers and neurons, as well as different activation functions, while we adopted default values for all the remaining parameters. To increase the network robustness, the weights were randomly initialized at every execution of the algorithm.

We have already mentioned the optimal architecture, for what concerns activation function, hyperbolic tangent was used. We performed extensive search for optimal values thanks to the ReCaS data center^6^; further details about the computational infrastructure are provided in Supplementary Materials. Thanks to cross-validation analysis we reached an optimal (and stable) configuration. In order to get a fair comparison with other regression models, we tried to use default configurations whenever possible; parameters whose values were tuned in cross-validation, as for example the number of trees in Random Forests, are explicitly mentioned, otherwise default values must be assumed.

#### Ridge Regression

Ridge regression (Hoerl and Kennard, 1970) is a substantial improvement of standard least square regression in those case where independent variables suffer or may suffer from multicollinearity. By definition, multicollinearity consists in the presence of high intercorrelations among the independent variables of the model; when present, multicollinearity can strongly affect the reliability of statistical inferences. Even if brain patches are sufficiently large to mitigate spatial correlations, it is not safe to assume, *a priori*, that neighbor patches are completely independent.

Ridge regression is basically a least square methods. Using the standard notation a regression equation is written in matrix form as *Y* = *Xβ*+*e* with *Y* the dependent variable, *X* the independent variables, β the regression coefficients, and *e* the residuals. Ridge regression prescribes, as standard linear regression, the minimization of the residual sum of squares (RSS):

$$\text{R}S\text{S}=\overset{S}{\underset{i=1}{\sum }}{\left(y_{i}-\beta_{0}-\overset{p}{\underset{j=1}{\sum }}\beta_{j}x_{ij}\right)}^{2}$$

where *S* is the sample size and *p* the number of independent variables. The difference with standard linear regression is that Ridge regression introduces a penalty or regularization term on the sum of squared coefficients:

$${\text{R}S\text{S}}_{Ridge}=\overset{S}{\underset{i=1}{\sum }}{\left(y_{i}-\beta_{0}-\overset{p}{\underset{j=1}{\sum }}\beta_{j}x_{ij}\right)}^{2}+\lambda \overset{p}{\underset{j=1}{\sum }}\beta_{j}^{2}$$

It is evident that when λ → 0 Ridge regression coincides with ordinary least square regression. When λ → ∞ the high regularization penalty makes some coefficients small, but yet not negligible, thus their effect is limited but still included in the model. Accordingly, the effectiveness of Ridge regression depends on the tuning of λ penalty: models with small λ values tend to have high variance and small bias, on the contrary high λ values involve small variance and high bias. For the present work, we explored several λ values in cross-validation.

#### Lasso Regression

Ridge regression considers any independent variable from the model whereas Lasso (Least absolute shrinkage and selection operator) regression (Tibshirani, 1996) tackles this issue allowing the exclusions of some coefficients. Accordingly, Lasso regression tries to retain the important features and discard those yielding a negligible contribution to the model.

Lasso residual sum of squares is similar to Ridge regression except for introducing as a penalty contribution the sum of the absolute values of the regression coefficients:

$${\text{R}S\text{S}}_{Lasso}=\overset{S}{\underset{i=1}{\sum }}{\left(y_{i}-\beta_{0}-\overset{p}{\underset{j=1}{\sum }}\beta_{j}x_{ij}\right)}^{2}+\lambda \overset{p}{\underset{j=1}{\sum }}|\beta_{j}|$$

Here, again, *S* is the sample size and *p* the number of independent variables. When λ → 0 Lasso regression coincides with the ordinary least square regression; when λ → ∞ Lasso tends to the null model with all coefficients β_*j*_ being 0 and the only non-vanishing value being the intercept. It is worth noting that, as for Ridge regression, Lasso regression needs the tuning of λ parameter in order to balance variance and bias of the model. As for Ridge regression, we explored several λ values in cross-validation.

#### Random Forest

**Figure 4:**
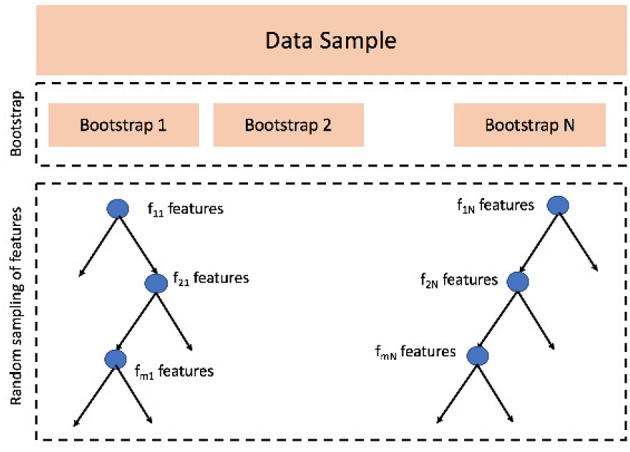
Random Forest algorithm consists of two main phases: (1) data sample is bootstrapped *N* times, *N* is the number of trees and the first parameter this algorithm needs to be set; (2) when growing a tree *j*, at each node splitting *i* a random set of features *f*_*ij*_ is sampled; the features selected change at every split, but the number of sampled features, the second parameter to be set, remains constant.

Another option for regression, extremely popular in recent years, consists in using ensemble learning. Among the possible choices, the most adopted and widely used algorithm is Random Forest (Liaw et al., 2002). Random Forests are constructed bootstrapping the data sample and growing a number of different regression trees, each of them using a different bootstrap, statistically with the original dataset. Besides, as a difference with bagging strategies, Random Forests add a further layer of randomness by growing each tree with a different set of predictors randomly selected every time a node is split, see Figure 4 for a schematic representation.

The main advantage of Random Forest over classical regression strategies is its robustness on overfitting; moreover, it is a good approach for preliminary investigations in the sense that, depending only two parameters, the number of trees to be grown and the number of features to pick at each node split, Random Forests is easy to tune and control.

A relevant aspect to consider is that Random Forest yields useful information about feature importance, thus resulting in interpretable models and a ranking about the association between each independent variable and the dependent variable, a crucial property in clinical applications. The Random Forest regression was tuned in cross-validation to search optimal values for the number of trees and the number of features to select.

#### Support Vector Machine

Finally, we evaluated the regression performance using Support Vector Machine (Smola and Schölkopf, 2004). Support Vector Machine regression is based on a well grounded statistical framework whose basic idea consists in using the available observations to learn a function *f*(*x*) that has deviations ϵ_*i*_ < ϵ from targets *y*_*i*_. As a consequence, the model learns to be accurate at least as the prescribed ϵ precision or, in other words, it does not accept deviations larger than ϵ. For clinical purposes this approach is of fundamental importance, as it guarantees the existence of a limit value which should not be exceeded for the validity of the model.

The main advantages of Support Vector Machine are 2-fold: (i) it is a versatile algorithm which can give accurate results in very different applications, comprising medical ones; (ii) it yields a compact representation even for huge datasets, thus it is a suitable choice for big data applications. The main drawback is probably the need to tune several parameters in order to achieve the perfect balance between variance and bias of the model. A not exhaustive list of parameters to tune include:

- the precision of the model ϵ;
- the kernel used for training and prediction, possible choices are: linear, polynomial (in this case one has to set the degree of the polynomial too), radial basis and sigmoid;
- the cost value for regularization;

Accordingly, for Support Vector Machines to be consistently effective it is fundamental to perform a wide search of the parameter space with a subsequent significant increase of the computational effort. Nevertheless, the use of modern data-centers can easily manage the needed requirements in terms of memory and processing time, thus the computational issues do not discourage the use of this learning framework. We explicitly explore the precision and the cost value for regularization.

## Results

###  Deep Learning Prediction Accuracy

**Figure 5:**
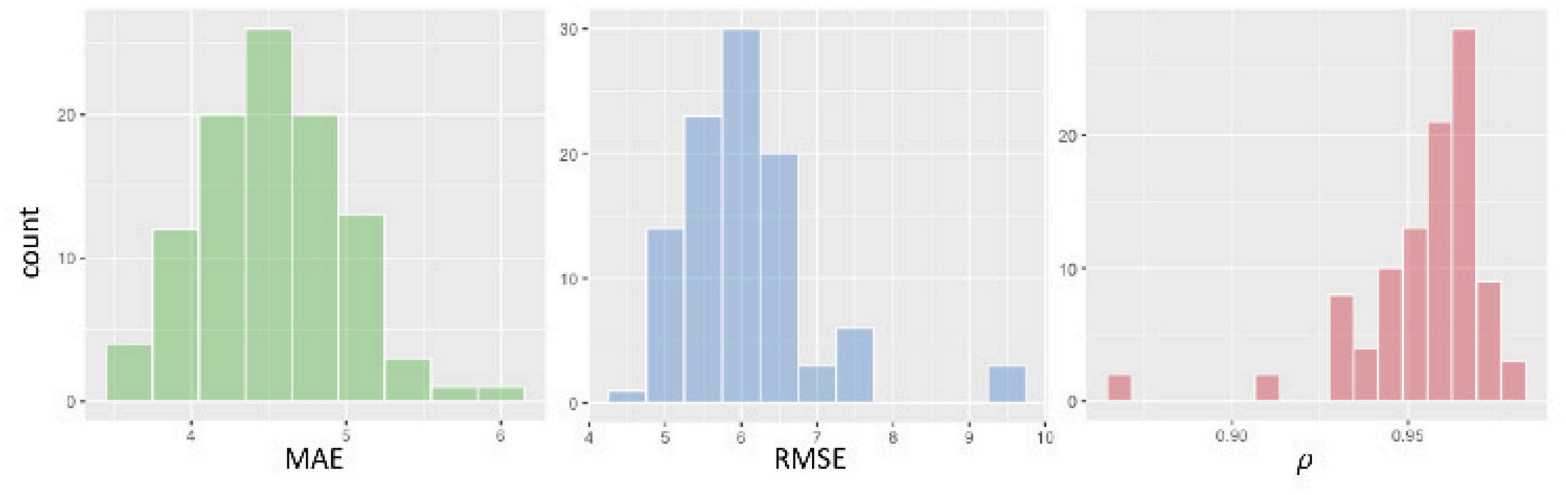
From left to right, histogram of cross-validation results: MAE, RMSE, and Pearson's correlation ρ.

We assessed the performance accuracy of our deep learning model by evaluating three distinct metrics: Mean Absolute Error (MAE), Root Mean Squared Error (RMSE), and Pearson's correlation ρ. The results presented in Figure 5 show the estimates of these metrics obtained with 100 rounds of 10-fold cross-validation.

Average MAE is 4.7 years, the MAE standard error is 0.1. For what concerns RMSE and correlation, our cross-validated estimates are: RMSE = 6.2±1.1 and ρ = 0.95±0.02.

A not secondary aspect to consider about the reliability of age-predicting models is their homoscedasticity either their heteroscedasticity. We performed the Breush-Pagan test to evaluate the presence or absence of heteroscedasticity and found *p* = 0.008, thus rejecting the null hypothesis, with 5% significance, for the variance of the residuals to be constant over the whole age range.

###  Age Ranges Affecting the Model Accuracy

**Figure 6:**
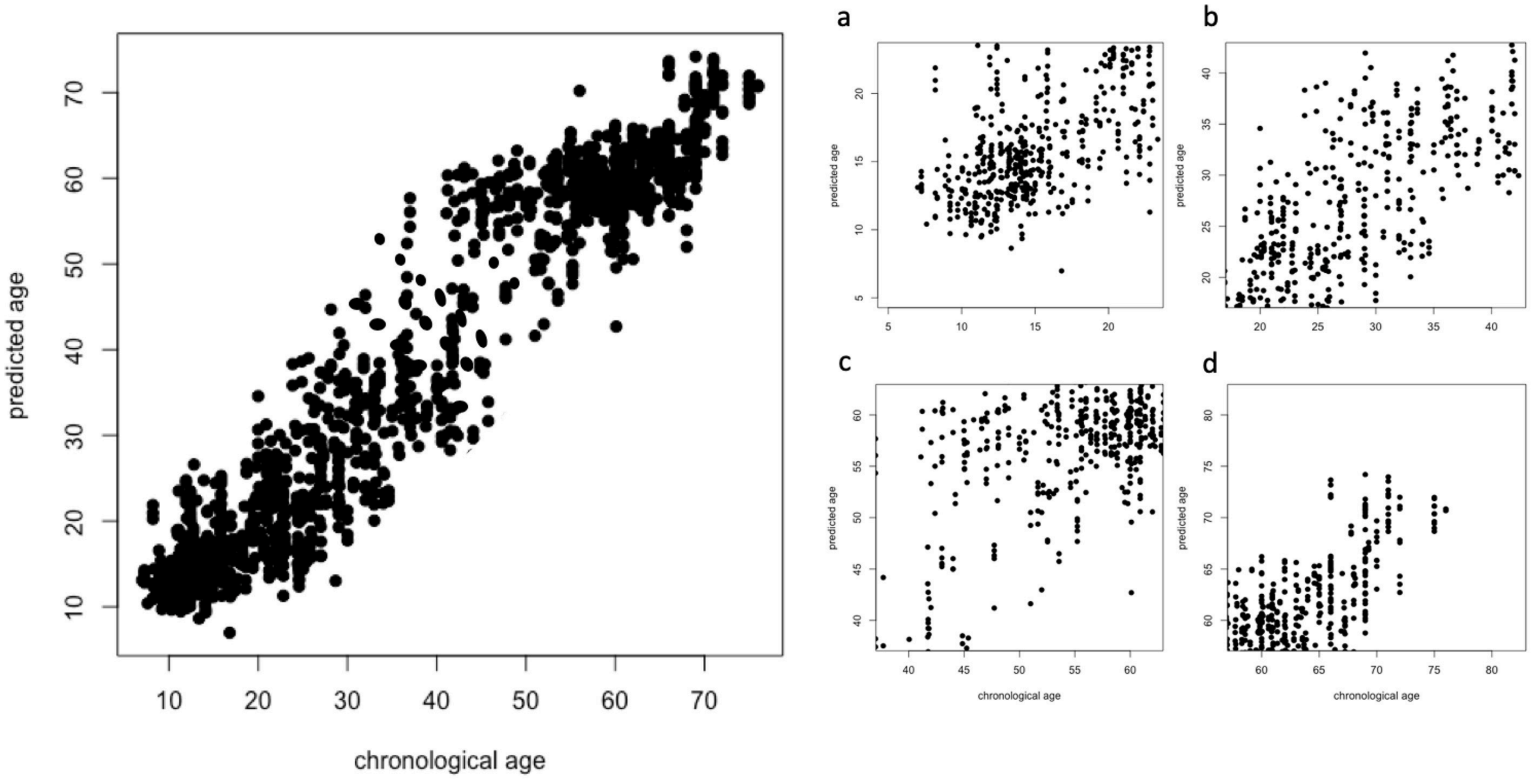
Overall scatter plot of chronological age (x-axis) and predicted age (y-axis) and the specific four age ranges (right panel): 7 ≤ Age < 20 **(A)**, 20 ≤ Age < 40 **(B)**, 40 ≤ Age < 60 **(C)**, 60 ≤ Age < 80 **(D)**.

To further investigate the effectiveness of our deep learning model, we evaluated if the regression accuracy was subject to significant changes when considering specific age ranges. In particular, see Figure 6 for the overall scatter plot (left panel) and four age ranges (right panel): 7 ≤ Age < 20 (a), 20 ≤ Age < 40 (b), 40 ≤ Age < 60 (c), 60 ≤ Age < 80 (d).

These distributions are significantly different according to a Kruskal-Wallis rank sum test (*p* < 2.2e^−16^); in particular, the best results are obtained for younger subjects while the performance has a significant drop when considering the groups including older subjects, see Table 1 for a comprehensive overview.

**Table 1:** Performance metrics obtained in different age ranges.

**Age range**	**MAE**	**RMSE**	**ρ**
7−20	**3.7 ± 0.2**	**3.9 ± 0.1**	0.43 ± 0.02
20−40	5.1 ± 0.2	6.6 ± 0.1	0.57 ± 0.01
40−60	6.5 ± 0.2	8.2 ± 0.2	**0.60 ± 0.01**
60−80	4.4 ± 0.2	6.6 ± 0.3	0.41 ± 0.03

*In particular, correlation, due to a drastic reduction of the sample size and range, suffers the highest reduction. Best values are in bold*.

Correlation is the metric suffering the highest drop in performance over all the considered age ranges. MAE and RMSE share a common behavior, their best values are found when age ranges from 7 to 20; the best correlation is found when 40 ≤ Age < 60.

###  Sample Size Effect

**Figure 7:**
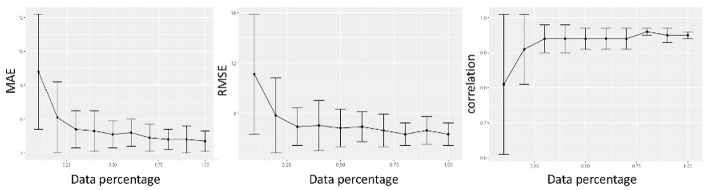
We evaluated the regression metrics MAE, RMSE, and correlation by randomly sampling a varying percentage of subjects from the whole cohort, from 10 to 100%, and reported the results of 100 ten-fold cross-validations.

Previous studies about age prediction using MRI have established the pivotal importance of sample size to obtain accurate age-prediction models. Accordingly, we present in Figure 7 the assessment of the sample size effect on the accuracy of our model.

The results are 2-fold: performance is affected by sample size, the more the available data, the more accurate age prediction; when using 80% of data, the deep model reaches a robust plateau. Whatever we considered, MAE, RMSE, or ρ correlation, the performance increased with the sample size, besides the variance of the model decreased.

###  Other Regression Strategies

To demonstrate the pivotal role of deep learning, we used the multiplex features to feed other state-of-the-art regression approaches. In particular, we compared deep learning with Ridge and Lasso regressions, Random Forest, and Support Vector Machine. Table 2 shows the comparison among best configurations, further details about parameter tuning and optimal values are reported in Supplementary Materials.

**Table 2:** Comparison of cross-validation regression performances for deep learning, Ridge and Lasso regression, Random Forest, and Support Vector Machine.

**Model**	**MAE**	**RMSE**	**ρ**
Deep learning	**4.7 ± 0.1**	**6.2 ± 1.1**	**0.95 ± 0.02**
Ridge regression	6.0 ± 0.7	7.8 ± 1.3	0.92 ± 0.03
Lasso regression	6.4 ± 0.7	8.2 ± 1.3	0.92 ± 0.03
Random forest	5.9 ± 0.7	7.6 ± 0.9	0.94 ± 0.02
Support vector machine	5.6 ± 0.7	7.2 ± 0.9	0.94 ± 0.01

*The reported values are those obtained after grid search for optimal configurations. Best results are presented in bold*.

Deep learning provides the most accurate model with respect of all the considered metrics. After deep learning, Support Vector Machine gets the best results, nonetheless, deep learning yields a significant increment of about 16% in terms of MAE and 14% in terms of RMSE. For what concerns correlations, even if providing the best performance, deep learning does not seem to significantly improve this metric, another clue suggesting the need for using correlations *cum grano salis*.

###  Feature Importance and Clinical Validation

To investigate which features had a strategic role in the age prediction, we calculated variable importances by using the Gedeon method (Gedeon, 1997) implemented in the “h2o” R package. This implementation considers the weights connecting the input features to the first two hidden layers and provides, for each features, the relative importance normalized between 0 and 1. We computed the importance ranking over different subject samples in order to select the most strategical features in terms of relative importance and occurrence. We obtained 113 features whose occurrence had not happened by chance (with a 5% comparison threshold with Bonferroni adjustment). In Table 3, the first 10 features, directly connected to a patch, are reported in order of mean relative importance along with the corresponding anatomical regions pinpointed by that patch.

**Table 3:** First 10 features in order of relative importance for aging prediction along with the related cortical and subcortical brain regions.

**Features**	**Patch**	**Mean relative importance**
Inverse participation	(L) Heschl's Gyrus (includes H1 and H2), Insular Cortex (GM, WM)	0.95
Multistrength	(L) Cingulate Gyrus, anterior division, Cingulate Gyrus, posterior division, Precentral Gyrus (GM)	0.89
Inverse participation	(L) Planum Polare, Heschl's Gyrus (includes H1 and H2), Central Opercular Cortex (GM)	0.89
Multistrength	(L) Frontal Pole, Frontal Orbital Cortex (GM)	0.89
Inverse participation	(R) Paracingulate Gyrus, Cingulate Gyrus, anterior division (GM, WM)	0.89
Strength	(L) Brain Stem, Parahippocampal Gyrus, posterior division (GM)	0.89
Inverse participation	(R) Precentral Gyrus, Post-central Gyrus (GM,WM)	0.88
Inverse participation	(L) Lateral Occipital Cortex, inferior division, Middle Temporal Gyrus, temporo-occipital part (GM, WM)	0.88
Inverse participation	(L) Lateral Occipital Cortex, superior division (GM)	0.88
Inverse participation	(L) Inferior Frontal Gyrus, pars opercularis, Precentral Gyrus, Middle Frontal Gyrus (GM, WM)	0.88

*(L) and (R) indicate left and right hemispheres; (GM) and (WM) indicate that gray and white matter are respectively included in the patch corresponding to a certain feature*.

**Figure 8:**
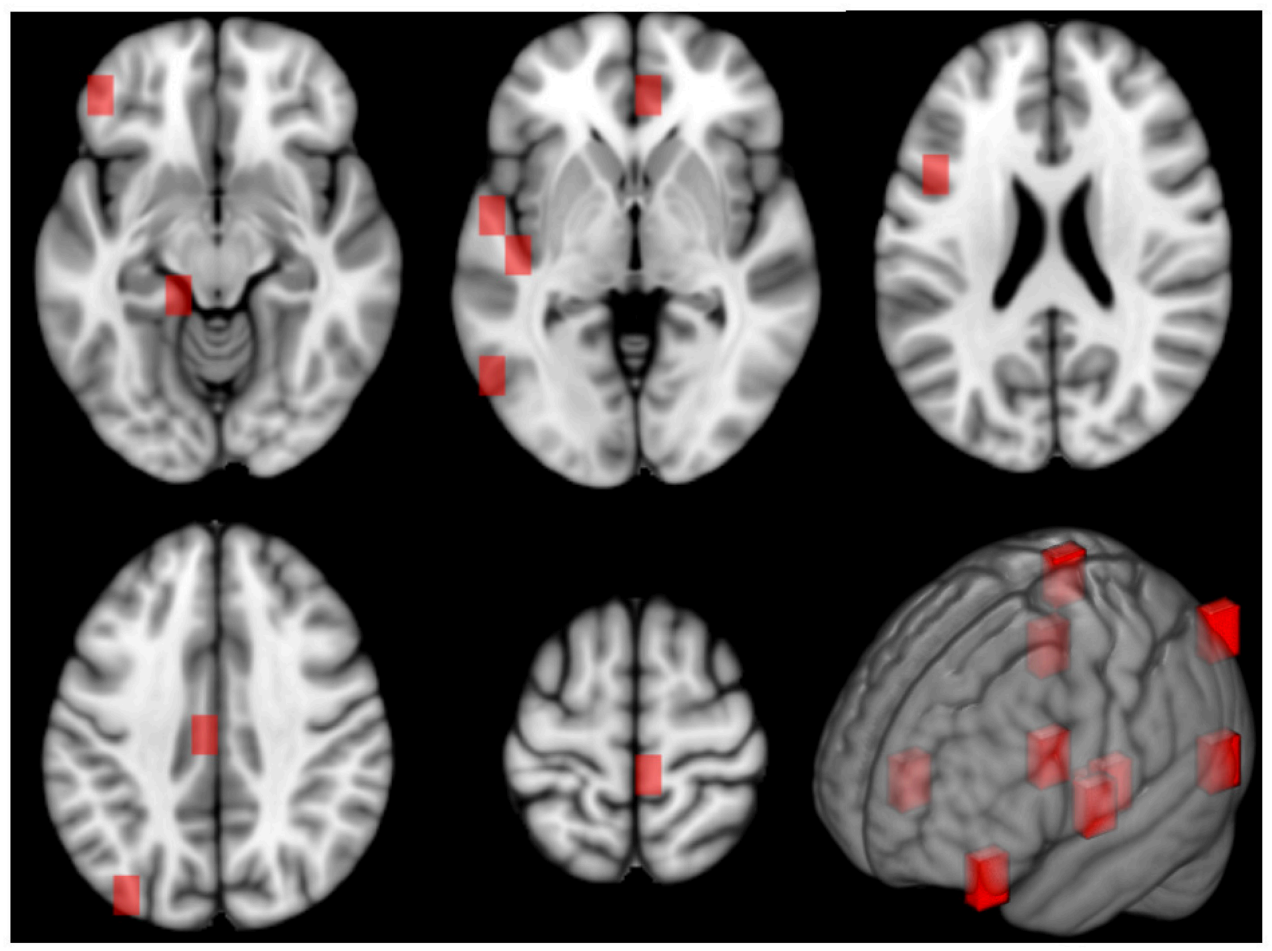
This figure shows the patches related to the first most important features along 5 axial planes of the MNI 152 template. On the bottom right, a 3D representation of the patches on the reference space is reported, as well.

The different cortical and sub-cortical anatomical regions, which are proved to be connected with aging, were found by mapping the related patches on the Harvard-Oxford atlas (Desikan et al., 2006). In Figure 8, the patches related to these anatomical regions are underlined in red on the MNI 152 template. It is worth to specify that these clinical findings are totally in agreement with the literature as argued in the Discussion section.

## Discussion

The method presented in this work, based on the multiplex model combined with a deep learning regression network allows the most accurate age prediction, in comparison with other standard machine learning approaches. Performances presented here compare well with results recently published (Franke et al., 2012; Cole et al., 2017a), including voxel-based approaches, provided the following considerations. First of all, the dataset used in this work is smaller than those investigated in the mentioned works; we have confirmed here that as the sample size increases predicting models tend to be more accurate and with less variance. Nevertheless, as the fraction of data employed exceeds 80%, improvements become significantly smaller; the deep learning model is robust and stable. A not secondary aspect to consider is age distribution: in this work we have analyzed a roughly uniform cohort, which is not the case, e.g., in Cole et al. (2017a). However, the dependence of performance on dataset composition/homogeneity certainly requires further investigation.

Another important aspect to consider about the general validity of the presented results concerns the image processing pipeline. In this study, we used the FSL library; FSL provides a consolidated and widespread tool for brain extraction. Nevertheless, other spatial normalization tools could be used, as for example SPM DARTEL a particularly suitable tool for normalization of elder subjects (Pereira et al., 2010). Actually, there is no general consensus indicating which tool should be preferred, on the contrary it is common for neuroimaging studies to define dedicated pipeline exploiting a wide range of existing tools, such as those previously mentioned, but also including FreeSurfer, ANTs and novel ones (Shen et al., 2013; Im et al., 2015; Hazlett et al., 2017).

In fact, we demonstrated here that age predictions are affected by heteroscedasticity; accordingly, a large data sample uniformly covering the lifespan range could mitigate this issue. Heteroscedasticity also affects performance accuracy: best performances in terms of MAE and RMSE are found for younger subjects (in the [7−20) range). This would confirm the necessity to compare age prediction accuracy declared in different studies with the caveat that age distribution of examined cohort should be consistent. This behavior suggests that morphological differences in healthy brains are accentuated in later years, younger brains tend to be less heterogeneous and, therefore, more adherent to a common pattern. However, it is worth noting that the extent of the age-range influences the MAE, with wider age-ranges yielding harder prediction problems; accordingly, we cannot conclude that the model performs better. This consideration about the influence of the age-range on the MAE is also important when comparing the current results between other studies.

Pediatric images usually require specific processing. Actually, children's brains significantly differ from the adult ones, because their growth is characterized by a series of non-linear changes occurring throughout the development ages; this is particularly true between 0 and 7 years. However, we do not expect this effect to significantly affect our analysis, because this specific range was not included in the analysis. Nonetheless, the standard pipeline adopted here is based on a template developed from adult brain data, which are not optimized for pediatric scans and, therefore, this could limit the accuracy of our model. In future work, we plan to focus on age prediction in younger cohorts, limiting the considered age range, and consider dedicated image processing strategies specifically tailored for younger subjects as suggested in recent works (Vân Phan et al., 2018).

A different consideration holds for correlation. Correlations are heavily affected by the overall range of the independent variable, when considering age sub-samples this range decreases, the number of observations decreases too; as a consequence, the resulting correlations do not match with the values computed using the whole dataset. On the other hand, the other metrics take into account only the relative difference between observed and predicted values. In other words, MAE and RMSE on average tend to reproduce in the age subsamples the same behavior they have on the entire dataset. This is not true for correlation. An interesting aspect to investigate in the future could be the assessment of which factors (sample size within each age range, multi-site effect on data heterogeneity, …) are mostly responsible for this issue. However, deep learning is by far the most accurate method to predict brain age, followed by Support Vector Machine. The intrinsic possibility to manage and model non-linear complex relationships offered by deep models seems to provide a significant advantage when attempting to predict brain age.

Another aspect investigated in this study was the feature importance aimed at finding out which features and which related anatomical regions were more accountable for the age prediction. We chose to not perform a dedicated feature selection in order to outline the role played by the different regression strategies. Of course, feature selection can play an important role in enhancing the performance of machine learning, nevertheless, the focus of this work was to establish the most effective strategy to exploit the informative content provided by our complex network model, independently from other processing steps.

It is interesting to notice as the most important features are often related to patches which identify several times the same anatomical regions demonstrating their prominent role in the aging process. Many studies report that these regions are widely involved in morphometric changes connected with age (Koini et al., 2018). Indeed, significant age-related reduction in cortical thickness, surface area, and volume have been found in areas like Heschl's gyrus, cingulate and paracingulate gyrus, parahippocampal gyrus, and temporal lobe which includes also the planum polare and Heschl's gyrus (Mann et al., 2011; Torii et al., 2012). These two latter regions play an important role in auditory processing which is notoriously affected by age advancement (Warrier et al., 2009). Cingulate and paracingulate gyrus are implicated in attention and emotional regulation, and parahippocampal gyrus and medial temporal lobe are involved in memory. Therefore, these regions also influence cognitive processes which are still connected with normal aging. A particular vulnerability to cortical thickness changes with age was seen in middle frontal gyrus, pre-central gyrus, post-central gyrus, and in the pars opercularis of the inferior frontal gyrus. The importance of frontal lobe regions is supported by evidence of age-related decline in several cognitive processes such as speed of processing, working memory, cognitive control, and motor control (Thambisetty et al., 2010; Lemaitre et al., 2012). Age-related changes have been also underlined in insula cortical thickness and in brain stem volume (Churchwell and Yurgelun-Todd, 2013; Lambert et al., 2013). However, the reader should take into account that the proposed approach defines a mathematical framework rather than a real biomedical brain network and it should not be overinterpreted

In our results, most of the regions related to the first 10 important features are located in the left hemisphere. This may suggest an age-related decrease or increase of correlation between the patches related to the important features in the left hemisphere and the others. Many studies report that structural and functional hemispheric asymmetry is related to age. Besides, changes in structural brain asymmetry with age have been found right in inferior frontal gyrus, anterior insula, anterior cingulate parahippocampal gyrus, and precentral gyrus (Kovalev et al., 2003), thus, in agreement with our results. Further investigations in this sense could be interesting also to examine a still open issue: whether and which hemisphere ages faster that currently is still an open issue (Esteves et al., 2018). However, the reader should take into account the proposed approach defines a mathematical framework rather than a real biomedical brain network and it should not be overinterpreted.

Finally, it is worth to mention an aspect that is gaining more and more interest, which is the increasingly widespread of “artificial intelligence” and machine learning for health purposes, especially for the development of diagnosis support systems. On one hand, thanks to deep learning there is the possibility to use raw data to directly predict age, height, or subject-specific clinical scores, the presence of pathological conditions and eventually their severity. On the other hand, thanks to particular inversion strategies, recent works have demonstrated the possibility to retrieve sensible information on patients even when using pre-trained models (Fredrikson et al., 2015). With this perspective, using our multiplex model, mediating between raw data and clinical score, in this case age prediction, could be also considered a safe way to use sensible data and protect the users' privacy, not to mention the computational advantage in terms of processing time.

## Conclusions

In this work, we demonstrated that: (i) the features retrieved with our novel brain network model can accurately characterize the normal aging, besides their informative content compares well with state-of-the-art; (ii) the informative power of multiplex features is effectively exploited and significantly maximized when using a deep learning regression. The proposed methodology localizes the brain regions most affecting aging in the left hemisphere. For what concerns the model accuracy, further investigations should be performed by increasing the sample size; the presented results are promising, nevertheless the statistical robustness of this study would greatly benefit from a larger dataset, besides this would be of paramount importance for a fair comparison with other studies. Finally, we observed here that brain aging is strongly affected by heteroscedasticity, this effect should properly taken into account by studies investigating lifespan processes; in particular, worst prediction accuracy was obtained in the age range 40−60, this would reflect the high specificity and variability characterizing brain atrophy in these years. Nevertheless, further investigations, exceeding the aims of the present work will be needed to corroborate such hypothesis.

## Ethics Statement

All experiments were performed with the informed consent of each participant or caregiver in line with the Code of Ethics of the World Medical Association (Declaration of Helsinki). Local institutional ethics committees approved the study.

## Author Contributions

NA designed the study and conceived the model. NA and ML performed statistical analyses. All authors interpreted the data, wrote, and approved the manuscript.

### Conflict of Interest Statement

The authors declare that the research was conducted in the absence of any commercial or financial relationships that could be construed as a potential conflict of interest.

## Footnotes

^1^http://fcon_1000.projects.nitrc.org/indi/abide/

^2^http://adni.loni.usc.edu

^3^http://fcon_1000.projects.nitrc.org/indi/retro/BeijingEnhanced.html

^4^https://ida.loni.usc.edu/

^5^https://brain-development.org/ixi-dataset/

^6^https://www.recas-bari.it/index.php/en/
