# Supplementary material for: Deep Learning and Multiplex Networks for Accurate Modeling of Brain Age
Source: Front Aging Neurosci. 2019 May 22;11:115. doi: 10.3389/fnagi.2019.00115 (PMC6538815; doi:10.3389/fnagi.2019.00115)
Supplement: Supplementary file 1 [file Presentation_1.pdf]

Supplementary Material

1 DEMOGRAPHIC INFORMATION

In the following Table S1 we provide an overview of the data used in this paper.

Table 1. Performance metrics obtained in different age ranges. In particular, correlation, due to a drastic reduction of the sample size and range, suffers the highest reduction. Best values are in bold.

Cohort	N	Age (range)	Sex (M/F)	Scanner
ABIDE	178	7-50	131/47	Various (all 3T)
ADNI	60	55-77	33/27	Various (1.5/3.0 T)
Beijing	60	18-26	23/37	Siemens (3T)
ICBM	58	30-60	28/30	Siemens (1.5T)
IXI	128	24-70	53/75	Various (1.5T)
**tot.**	**484**	**7-80**	**268/216**	

2 PARAMETER CONFIGURATIONS FOR REGRESSION MODELS

In this section, we provide the study of several parameters affecting the performance of the regression models evaluated in this paper.

2.1 Ridge regression

The most important parameter for Ridge regression is the cost λ. We present the results in the following Figure S1.

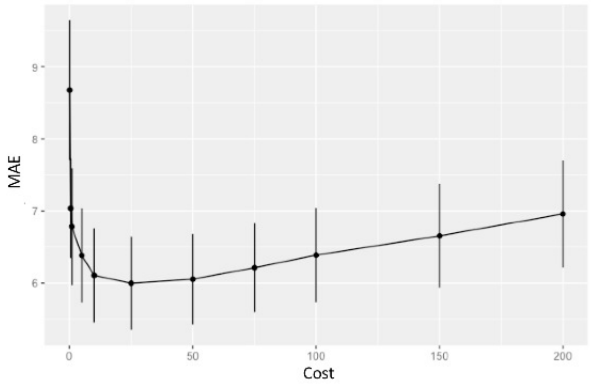

Figure S1. Best results are found for $\lambda = 25$.

Ridge regression has its optimal performance for $\lambda = 25$. A similar trend is shown for RMSE and correlation.

2.2 Lasso regression

Even in this case, cost is the most important parameter to investigate, see Figure S2

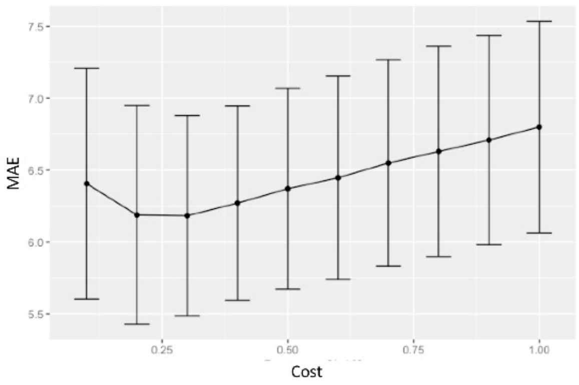

Figure S2. Optimal configuration is found for $\lambda = 0.3$.

Optimal configuration is obtained when $\lambda = 0.3$.

2.3 Random Forest

Random Forest algorithm depends on two parameters, the number of trees and the number of sampled features. We preliminary observed that 100 trees were sufficient to obtain a robust model. Then we investigated the optimal number of features, results are presented in the following Figure S3

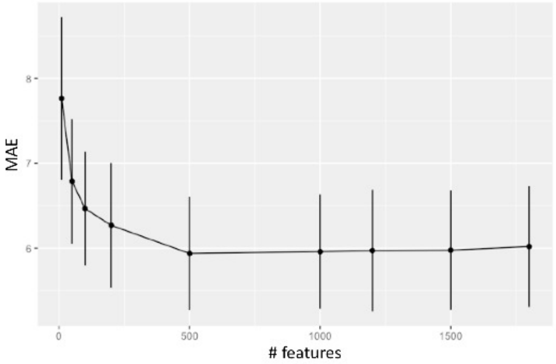

Figure S3. Best performance is obtained by sampling 500 features at each node split.

Best performance is obtained with 500 features.

2.4 Support Vector Machine

We investigated the regularization cost for Support Vector Machine. Results are presented in Figure S4.

Optimal configuration of Support Vector Machine requires regularization cost $C = 10$.

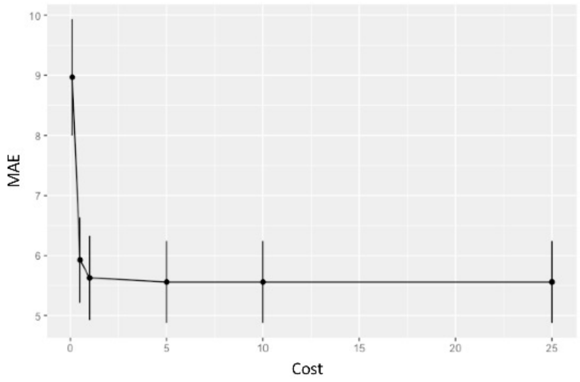

Figure S4. Optimal regularization value is $C = 10$.

3 COMPUTATIONAL INFRASTRUCTURE

The presented analyses were performed thanks to the Bari ReCaS datacenter. ReCaS was built by the University of Bari “Aldo Moro” and the National Institute of Nuclear Physics (INFN) in the framework of the ReCaS project (PON Research and Competitiveness $2007 - 2013$ Notice 254 / Ric). It was completed in July 2015 and inaugurated on July 9, 2015.

The aim of the ReCaS project was the upgrade of four datacenters in Southern Italy, namely Catania, Cosenza, Naples and Bari to provide the scientific communities, the public administrations and both public and private companies of these regions a computational infrastructure able to support big data analytics.

Since 2009, the “Bari Computing Centre for Science (Bc2S)” has supported the multi-disciplinary scientific activities of Bari researchers, ranging from high energy physics to space technologies, from computational physics to medical physics.

The ReCaS computing farm can count on a computing power of 12000 cores distributed over 400 nodes and about 5000 TB of shared disk space. In addition, the Recas datacenter houses a small cluster dedicated to High Performance Computing (HPC). The HPC cluster consists of 20 servers with 40 cores, for a total of 800 cores; each server is equipped with a graphics accelerator NVIDIA K40.
